# Different chest HRCT scan protocols change the extent of ground glass opacities

**DOI:** 10.1186/s12890-022-02212-7

**Published:** 2022-11-21

**Authors:** Össur Ingi Emilsson, Angelica Dessle, Henrik Johansson, Shamisa Adeli, Andrei Malinovschi, Maija-Leena Eloranta, Tomas Hansen

**Affiliations:** 1 Department of Medical Sciences, Respiratory, allergy and sleep research, Akademiska Sjukhuset, Uppsala University, 751 85 Uppsala, Sweden; 2 Respiratory medicine and allergology, Akademiska sjukhuset, Uppsala, Sweden; 3 Institution of Surgical Sciences, Department of Radiology, Uppsala University, Uppsala, Sweden; 4 Department of Medical Sciences, Clinical Physiology, Uppsala University, Uppsala, Sweden; 5 Department of Women’s and Children’s Health, Physiotherapy, Uppsala University, Uppsala, Sweden; 6 Department of Medical Sciences, Rheumatology, Uppsala University, Uppsala, Sweden

**Keywords:** High-resolution computed tomography, Interstitial lung disease, Warrick scoring, Positive expiratory pressure, Prone scan, Supine scan, Atelectasis

## Abstract

**Background:**

Ground glass opacity (GGO) is the main HRCT feature representing alveolitis in systemic sclerosis-associated interstitial lung disease (SSc-ILD), but may also represent other conditions such as atelectasis or edema. It is unclear how much this is affected by the HRCT scan protocol used. We aimed to compare the performance of three different HRCT protocols to evaluate the degree of SSc-ILD related changes.

**Methods:**

Eleven patients with SSc underwent chest HRCT scan by three different protocols: First, a supine scan after lying down for 15 minutes, then two scans in alternating order: A prone position scan, and a supine position scan after performing 10 deep breaths using a positive expiratory pressure (PEP) device. The HRCT scans were evaluated by the Warrick score system for ILD-related findings.

**Results:**

The three HRCT protocols were compared and resulted in different mean (95% CI) Warrick scores: 9.4 (5.3–13.4) in supine after rest; 7.5 (95% CI 3.8–11.1) in prone and 7.6 (95% CI 4.2–11.1) in supine after PEP. When comparing supine after rest to prone and supine after PEP, the latter two scans had a significantly lower score (*p* = 0.001 for both comparisons). In all cases, only sub-scores for ground glass opacities differed, while sub-scores for fibrosis-related changes did not change.

**Conclusions:**

Different HRCT scan protocols significantly altered the Warrick severity score for SSc-ILD findings, primarily because of changes in ground glass opacities. These differences may be clinically meaningful.

## Take-home message

HRCT scan protocols, differing in position or pre-scan breathing techniques, can significantly alter the extent of ground glass opacities, which may alter interpretation. Standardized HRCT protocols are important for precise clinical interpretations.

## Introduction

Systemic sclerosis (SSc) is a connective tissue disease clinically characterized by skin fibrosis and involvement of visceral organs such as lung, kidney and the cardiovascular system [1, 2]. Interstitial lung disease (ILD) is a common manifestation of visceral involvement. The reported prevalence of ILD, depending on the criteria used, varies from around 20–70% of the patients, and is one of the leading causes of mortality in SSc [3–5]. High-resolution computed tomography (HRCT) is the reference standard for assessing the presence and severity of SSc-ILD, and is found to be superior to conventional chest x-ray or ultrasound [6, 7].

The lung involvement in SSc-ILD includes alveolitis in early stages and irreversible fibrosis in more severe cases [8]. Ground glass opacity (GGO) is the HRCT feature representing alveolitis and can be the first signs of ILD or disease activity in an already known ILD [9–11]. However, GGO can also represent atelectasis due to gravitational forces, edema, or beginning fibrosis [12–14]. In the absence of additional more specific etiological findings, the interpretation can therefore sometimes be challenging. As the presence and extent of HRCT findings are important for the clinical judgement and choice of therapy [3, 15], it is important to determine whether the GGO represent active alveolitis or alternative causes such as atelectasis.

Different grading systems have been proposed for scoring the severity of SSc-ILD [9, 13]. One of the well validated and commonly used in research is the Warrick scoring system, were each lung segment is evaluated for typical ILD features giving an both a severity score and an extent score [9, 16].

When performing chest HRCT in supine position it is often noted that dependent parts of the lungs may exhibit increased opacity, often in a linear pattern similar to GGO indicating active alveolitis. SSc-ILD correlated active alveolitis is predominantly seen as GGO localized in lower lung lobes [17]. As GGO due to gravitational forces are also usually localized in the lower parts of the lung, it can in some cases be hard to distinguish GGO from gravitational forces from GGO representing active alveolitis [3]. To better differentiate GGO due to gravitational forces from GGO representing active alveolitis, some centres add an additional prone CT scan to their regular chest HRCT protocol, but this is still only implemented to a limited extent in both clinical practice and clinical studies, in part because of the increased radiation exposure [8, 18–20]. Studies specifically evaluating this protocol or other modes to optimize technique to conduct chest HRCT scans are scarce.

Theoretically, breathing manoeuvres might diminish atelectasis found on chest HRCT scans and therefore reduce the effect of the atelectasis component on GGO. Positive expiratory pressure (PEP) breathing is a non-invasive clinical manoeuvre commonly used to prevent or reverse atelectasis by increasing lung volumes [21–23].

The aim of this study was to evaluate the performance of different protocols for HRCT of the chest to evaluate the presence of ILD in SSc patients, and the severity grading of lung involvement expressed as a Warrick score. The methods studied were HRCT in the supine- and prone position, and a supine HRCT after PEP breathing.

## Materials and methods

### Study population

Eleven patients fulfilling the American College of Rheumatology classification criteria for SSc [24], were invited to participate in the study during their regular visit at the outpatient rheumatology clinic at Uppsala University Hospital, Uppsala, Sweden (Table 1). To minimize unnecessary radiation, only patients without a recent HRCT with a clinical indication for HRCT (screening, follow-up, symptom evaluation) were included in the study. Participants then underwent HRCT using three different protocols, further described below, thereby each patient being their own control, minimizing risk for unmeasured confounding. Data on patient characteristics (age, gender, BMI, smoking status, disease information including diffuse or limited disease, and lung function) were extracted from clinical data. The study was approved by the Swedish Ethical Review Authority (2019–02426) and written informed consent was obtained from all participants. All methods were carried out in accordance with relevant guidelines and regulations.

**Table 1:** Patient characteristics

Number of patients	11
Age, years (median (range))	62 (50–77)
Female/male gender ratio	10/1
BMI (median (range))	24.4 (19.5–33.6)
Current smoker	0
Diffuse disease	2
Years since diagnosis (median (range))	10.6 (0.9–47.1)
Total lung capacity (% of expected, median (range))	91% (49–115%)

### Chest HRCT protocol

The 11 participants underwent a HRCT scan of the chest upon inclusion in the study, consisting of three sequential HRCT scans of the chest, 1) supine scan after rest, 2) supine scan after PEP breathing (hereafter referred to as “post PEP scan”) and 3) prone scan. All scans were performed arms over head and with instructions for deep inspiration. No contrast agent was applied during the CT scan.

The first CT scan was always the supine scan after rest, after the participants had rested in a supine position for at least 15 minutes. This protocol was an attempt to maximize the position-dependent changes in the dependent parts of the lungs, and is representative for patients in hospital wards. After the rest, the participants got up and walked into the study room, received standardized instructions, and then lay down in the CT scanner.

The order of the two following scans alternated with every other participant to be able to control for possible variations due to the order of scans. The scans in prone position and post PEP were performed as sequential scans on the lower part of the chest, from carina and below. The rationale for this limited coverage was limitations in the amount of ionizing radiation in the ethical approval.

The following scan parameters were used on CT scanner (Siemens Somatom Definition Flash, Erlangen, Germany) for the deep inspiration supine scans; 90 ref. mAs, 120 kVp, slider pos 3, rotation time 0.5 sec, spiral scan with pitch 1.0. The inspiratory prone scan with; 110 ref. mAs, 120 kVp rotation time 0.33 sec, collimation 1 × 2 mm with feed 6 mm in a sequential scan. For the inspiratory scan after PEP; 110 ref. mAs, 120 kVp, rotation time 0.33 sec, collimation 1 × 2 mm with feed 6 mm in a sequential scan. Caredose 4D with were used for all scans. The images were reconstructed with Kernel Bi57 and reviewed with center − 500 HU and window 1500 HU.

### PEP breathing

The PEP-breathing was performed with a PEP device consisting of a T-piece with a one way valve and a mouthpiece (Intersurgical LTD, Workingham, Berkshire, UK). A resistance nipple, with a small lumen diameter connected to the device, created the flow resistance during expiration with a mid- expiratory pressure of approximately 10–15cmH_2_O. The participants were instructed to inspire as deep as possible, and then expire through the PEP device during 10 consecutive breaths. To minimize the risk of airway closure, the participants were instructed to end the expiration before emptying the lungs. The PEP-breathing was done with the participants in a sitting upright or standing position.

### Image evaluation

The scans were evaluated according to the previously described Warrick system, which is semi-quantitative scoring method that combines the severity of ILD features and the extent of the disease. The different abnormalities result in a different score depending on how severe the abnormality is [9]. GGO was defined by increased parenchymal density without obscuration of underlying lung architecture [25].

The abnormalities reported were: GGO (1p), irregular pleura margin (2p), septal or subpleural lines (3p), honeycombing (4p) and subpleural cysts (5p), resulting in a maximal severity score of 15p. The extent score is determined based on total number of segments involved with every one of the 5 different abnormalities described above (in total 18 segments, list of segments analysed is reported in Table 3). 1 to 3 segments involved gives to 1 p, 4 to 9 segments involved gives 2p, > 9 segments involved gives in 3p. Therefore the maximum extent score can be up to 15p, and added to the severity score can give a total Warrick score of 30p [9]. A Warrick score was calculated for the supine, the prone and finally the post PEP scan.

Two radiologists scored the images individually initially; one consultant specialized in thoracic radiology (TH) with 13 years of experience and one senior radiology resident (AD) with 4 years of experience. The total Warrick scores achieved good interrater agreement (Kappa-statistic 0.72 (Std. Err. 0.10)). The final reported score was then reached in consensus by the two radiologists. Both radiologists were blinded for clinical and research data to minimize risk for bias.

After scoring all studies, the radiologists gave a subjective evaluation as to which images or combination of images they preferred for clinical assessment purposes.

### Statistical analysis

The statistical software STATA 16.1 (StataCorp, College Station, TX, USA) was used for statistical analysis. Because of the low number of participants, Wilcoxon signed-rank test was used to compare Warrick scores between different HRCT scan protocols. The Warrick scores for this comparison were based on analysis of only the segments depicted in all HRCT scan protocols. A *p*-value < 0.05 was considered statistically significant.

## Results

Of the eleven participants, one was male, and the median age was 62 years. None was underweight, and none had obesity class 2 or higher (BMI > 35). Nine had a limited disease, and two had a diffuse disease. The range of years since diagnosis was wide, from one to 47 years (Table 1). Only one participant had total lung capacity below 80% of expected.

### Warrick score results

**Fig. 1:**
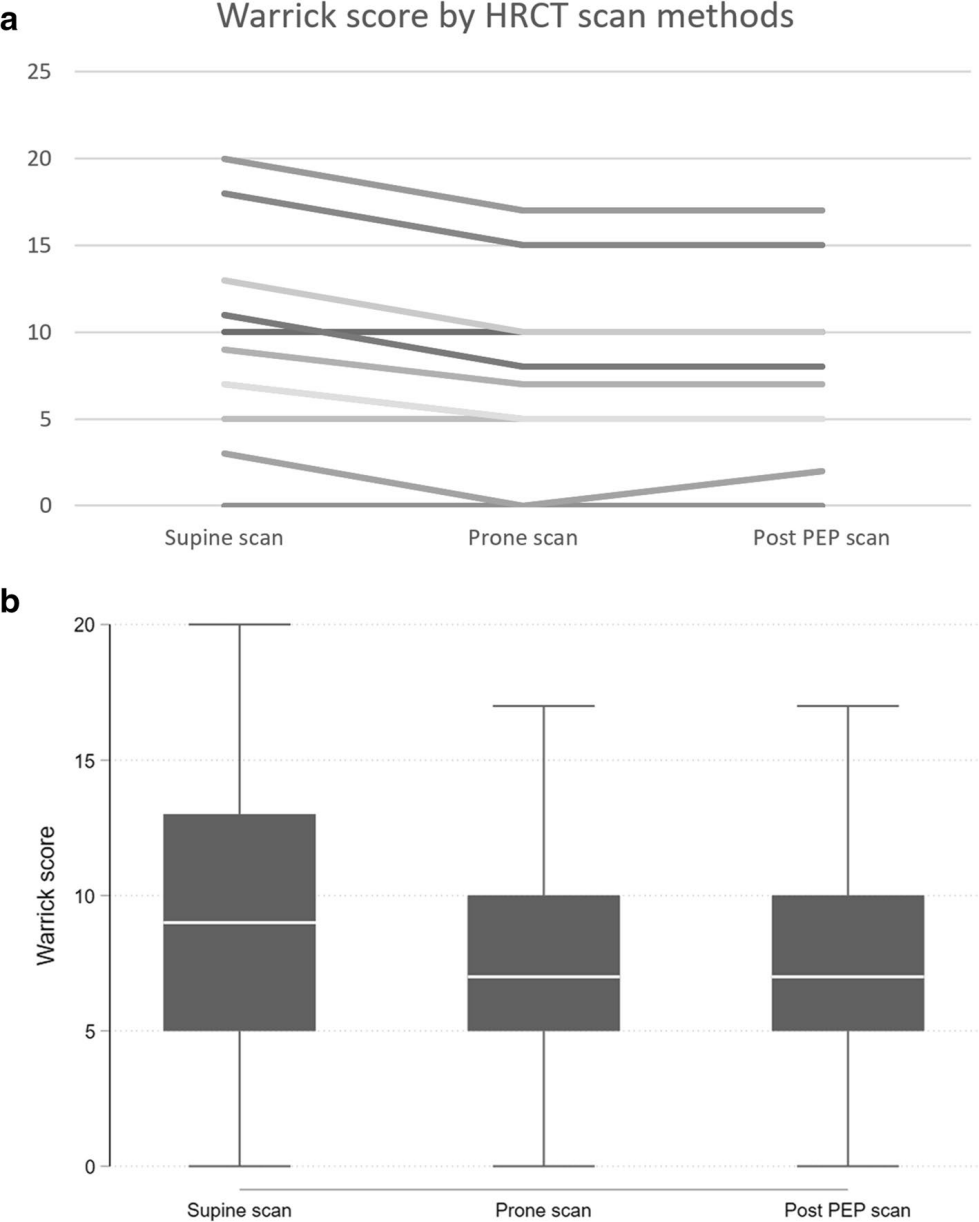
**a** Total Warrick score for all participants by scan method. Two participants had identical Warrick scores on all scans (score 7 on supine, 5 on prone, 5 on post-positive expiratory pressure (PEP)). Scores for the prone scans and post-PEP scans were significantly lower compared to the supine scans, but no statistically significant difference was seen between prone scans and post-PEP scans. **b** Data presented as a box-and-whisker graph

Figure 1 summarizes the total Warrick scores for all participants. The median value total Warrick score from the supine scans was 9 (IQR 5–13), the prone scans 7 (IQR 5–10), and the post PEP scans 7 (IQR 5–10). The prone scans and post PEP scans were significantly lower than the supine scans (*p* = 0.001 for both comparisons). None of the 11 participants had a higher Warrick score on the prone or post PEP scans than on the supine scan. The differences between the supine, prone and post PEP scans were solely based on changes in the extent of GGO (Table 2), whereas other aspects were unchanged (irregular pleura margin, septal or subpleural lines, honeycombing, subpleural cysts).

**Table 2:** Number of segments with ground glass opacities on HRCT scans

Participant no.	Supine after rest		Prone		Supine after PEP	
	Segments involved	GGO severity score	Segments involved	GGO severity score	Segments involved	GGO severity score
1	1	1	0	0	0	0
2	2	1	0	0	0	0
3	3	1	0	0	0	0
4	4	2	0	0	1	1
5	5	2	0	0	0	0
6	7	2	0	0	0	0
7	8	2	0	0	0	0
8	8	2	0	0	0	0

(Participants with no ground glass opacities excluded, *n* = 3)

Eight of the eleven participants (73%) had any degree of GGO in supine position. Thereof, three had a severity score of 1, and five had a severity score of 2. For one participant, GGO was still present in one lung segment on the post PEP scan (compared with four segments on the supine scan and none on the prone scan). For all other participants, no GGO was seen on the prone and post PEP scans.

### Radiologic evaluation

**Fig. 2:**
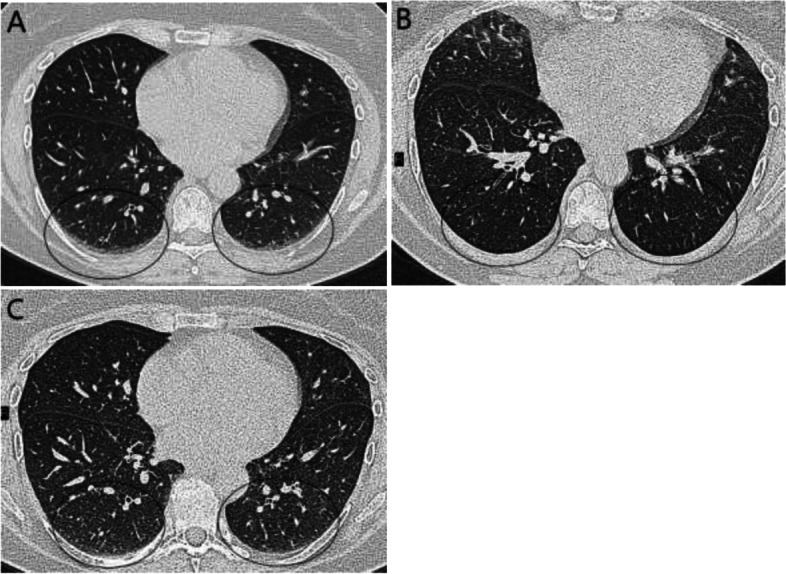
**A** A participant in supine position with ground glass opacities (GGO) in typical localization for atelectasis in the lower parts of the lower lobes indicated with circles. **B** Same participant in prone position showing no GGO. **C** Same participant post-positive expiratory pressure (PEP) showing no typical GGO. **B** and **C** resulted in the same Warrick score but with less opacification in the lower parts of the lower lobes in B as compared to C. (All slices representing the same lung area by ocular evaluation)

**Fig. 3:**
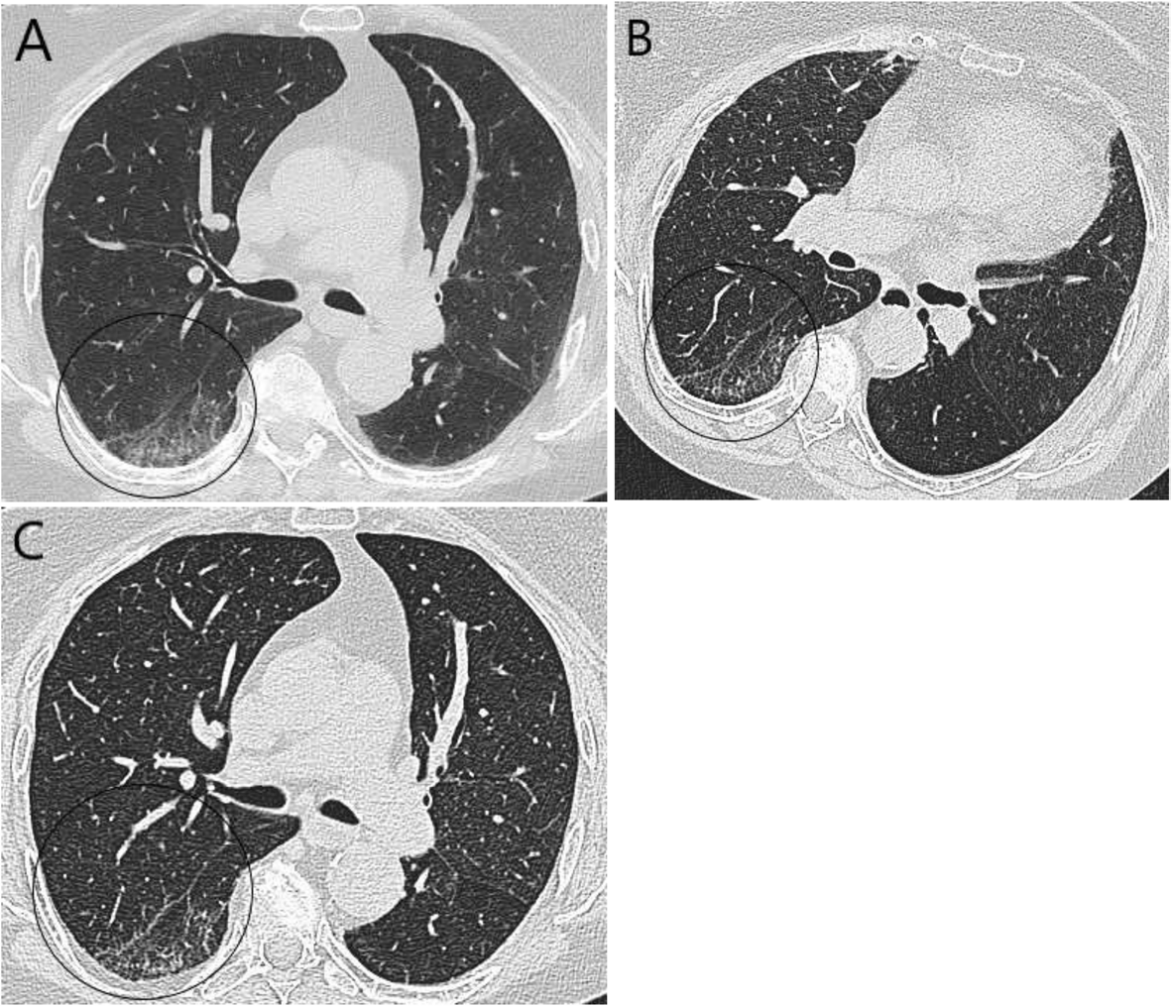
**A** A participant in supine position with ground glass opacities (GGO) in right lower lobe indicated with a circle. **B** Same participant in prone position showing no GGO in the same area but persistent subpleural lines. **C** Same participant post-positive expiratory pressure (PEP) showing similar findings with no GGO but persistent subpleural lines as in the prone position. (All slices representing the same lung area by ocular evaluation)

**Fig. 4:**
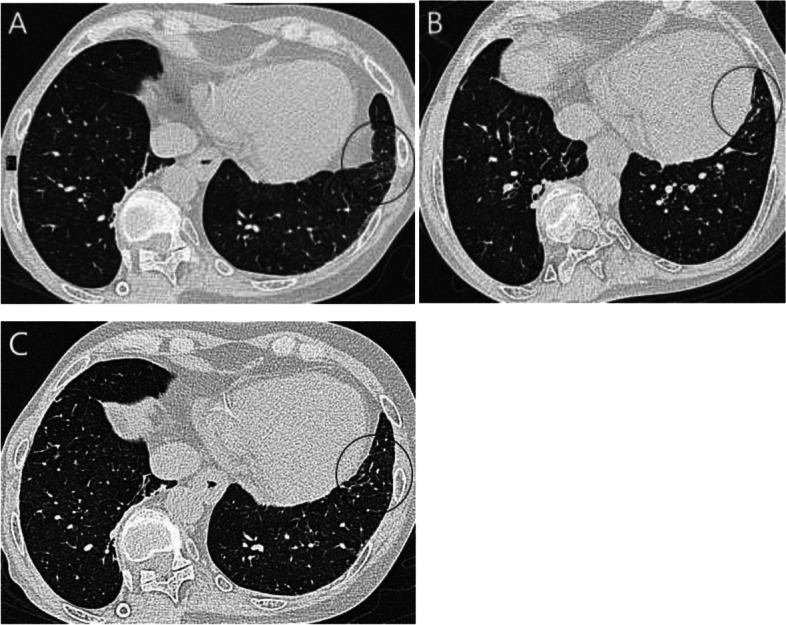
**A** A participant in supine position with ground glass opacities (GGO) in another typical localization in the lingular segment of left upper lobe, indicated with a circle. **B** Same participant in prone position, no GGO were present in the same area. **C** Same participant post-positive expiratory pressure (PEP), also no GGO were present in the same area. (All slices representing the same lung area by ocular evaluation)

The lobes most often affected were the right lower lobe (lateral and posterior segments) and left lower lobe (anteromedial, lateral and posterior segments) (Table 3). Examples of changes in GGO between scan protocols in the dorsal parts of the lungs, changes affecting the Warrick score, are presented in Fig. 2. On the prone scan, no GGO were seen, while on post PEP scan a persistent GGO was seen in one segment (Fig. 2). Figure 3 shows opacities assessed as GGO and septal or subpleural lines on the supine scan, but only septal or subpleural lines without GGO on the prone scan and post PEP scan. The prone scan and post-PEP scans were in this case judged equally good and resulted in the same Warrick score. Figure 4 displays GGO in lingula on the supine scan, but no GGO on the prone and post PEP scans, showing that also GGO not located in the lower parts of the lung decreased in the latter two scans.

**Table 3:** Number of participants with notable changes on supine scan by lung segments (n/total participants)

*Right upper lobe*	
0/11	Apical
0/11	Posterior
0/11	Anterior
*Middle lobe*	
1/11	Lateral
1/11	Medial
*Right lower lobe*	
2/11	Superior
0/11	Medial
2/11	Anterior
7/11	Lateral
7/11	Posterior
*Left upper lobe*	
0/11	Apicoposterior
1/11	Anterior
3/11	Superior lingular
0/11	Inferior lingular
*Left lower lobe*	
0/11	Superior
4/11	Anteromedial
4/11	Lateral
4/11	Posterior

## Discussion

In this study, we found that HRCT scans performed in a prone position or supine position after PEP breathing consistently altered the presence and severity of ILD-related findings in patients with systemic sclerosis, as compared with HRCT in the supine position after rest. This was mainly based on changes in ground glass opacities.

The majority of SSc patients in our study had GGO on a supine scan after rest, but as all GGO was absent on the subsequent scans (except for one post-PEP scan where minimal GGO remained), these were all judged to most likely represent small atelectasis, rather than a SSc-ILD related finding such as alveolitis. Even though the number of participants was low, the fact that each participant was their own control and that the pattern of results was highly consistent between participants, it is unlikely that the results are biased from small sample size.

GGO is a common finding in HRCT studies in the majority of SSc patients [26]. GGO in SSc-ILD has been associated with alveolitis and disease activity [10, 11], and may reflect a potentially reversible inflammatory manifestation in SSc-ILD in the absence of overt fibrosis [19, 27]. The extent and severity of parenchymal disease shown by HRCT including GGO is clinically relevant and correlates with the severity of pulmonary impairment and lung function tests [28]. The recognition of the different patterns may therefore be useful in targeting patients that can benefit from therapy, since GGO pattern could represent disease activity that can be reversible with treatment unlike cases with manifest fibrosis [26, 29–32]. Indeed, large clinical studies on immunosuppressive therapies have had GGO as inclusion criteria [33–35]. In light of this it is important to detect and quantify GGO reliably, as it may influence clinical management, but as our study shows GGO can be a protocol-related finding.

Few studies report on the minimal clinically meaningful difference in the Warrick score, but one study found that patients with a clinically judged progress in SSc-ILD had an increase in Warrick score by slightly more than one point on average [16]. Additionally, studies implementing artificial intelligence when analyzing HRCT are accumulating, where artificial intelligence seems to react to more subtle differences than traditional scoring systems [16, 20]. In our study, we found a difference of 2–3 points among 8 out of 11 participants. Such differences, if occurring between two separate visits, might thus lead to treatment changes. This further underscores the importance of noting the scan position, and possibly also protocols before the scan, as differences in scan methods may generate differences in results perceived as clinically meaningful.

The PEP protocol performed in this study, mainly aimed to reduce small atelectasis, was simple and quick to apply. The same effect may perhaps be achieved by deep breathing manoeuvres without a PEP device, but this was not evaluated in our study. In light of this, the difference between the scans performed in a supine position either after rest or after PEP breathing is notable. The significant difference seen in two supine scans with different pre-scan protocols underlines the importance of performing chest HRCT scans in truly deep inspiration. This is even more important in cases where prone scans are not feasible.

The difference noted between the protocols in this study does have a clinical implication. As previously mentioned, some centres perform a supine scan and then an additional prone scan to minimise risk for false interpretation of GGO findings [8, 18–20]. However, this is time consuming and increases radiation exposure. To achieve both time efficiency and limit the radiation exposure while maintaining reliable results, the results from this study would recommend a single supine post PEP scan instead of a supine after rest scan, as the supine post PEP scan decreases the magnitude of false positive GGO findings. Alternatively, if the PEP breathing technique may be difficult to implement, a similar effect may be achieved by deep breathing techniques without PEP [36].

Numerous previous studies on SSc patients implementing HRCT scan of the chest do not state how the scan was performed, including if the assessment was based on supine or prone positions or both [18–20]. In light of our findings, it is important to note the method of scanning, and evaluate the results accordingly.

The main strengths of this study are the high quality of HRCT scans performed, well-defined patient population, and thorough methodology regarding the different scan protocols. Furthermore, the staff performing the HRCT scans were trained by a registered respiratory physiotherapist on how to instruct participants how to perform the PEP breathing. However, some limitations occur as well in this study. First, the number of participants is low, but the magnitude of the difference was relatively large and statistically significant despite the low number of included participants. Second, for the prone and post PEP scan the entire lungs were not covered due to radiation aspects, including only the lung parenchyma from the carina and below. On the other hand, only one segment was affected in a whole-lung HRCT in the supine position but not covered in the two later scans. Therefore, nearly all pathology was evaluable by the three scan methods. Third, this study only recruited patients with systemic sclerosis, limiting the generalizability of the results. However, the processes responsible for the fleeting ground glass opacities should be similar in different patient groups, as they are likely not disease-specific but rather related to atelectasis.

## Conclusion

Our study demonstrates that both prone and after PEP breathing chest HRCT scan could alter the presence and degree of ground glass opacities in patients with systemic sclerosis, especially regarding ground glass opacities. The extent of the difference is in some cases considerable and might influence clinical decisions, which needs to be evaluated in future studies. We suggest that when performing chest HRCT scan, the method needs to be clearly described and preferably performed using methods that minimize risk of false-positive ground glass opacities, both in clinical practice and research.

## Data Availability

The datasets generated and/or analysed during the current study are not publicly available due the study being currently ongoing, but are available from the corresponding author on reasonable request.

## Abbreviations

SSc: Systemic sclerosis
ILD: Interstitial lung disease
HRCT: High-resolution computed tomography
GGO: Ground glass opacity
PEP: Positive expiratory pressure
BMI: Body mass index
